# The Effect of ThyroidߚStimulating Hormone on Stage of Differentiated Thyroid Carcinoma

**DOI:** 10.1002/edm2.266

**Published:** 2021-06-07

**Authors:** Laya Soleimanisardoo, Mohsen Rouhani, Fatemeh Soleymani Sardoo, Mohammad Hossein Gozashti

**Affiliations:** ^1^ Endocrine and Metabolism Research Center Institute of Basic and Clinical Physiology Sciences Kerman University of Medical Sciences Kerman Iran; ^2^ Pathology and Stem Cell Research Center Afzalipour Hospital Kerman University of Medical Sciences Kerman Iran

**Keywords:** differentiated thyroid cancer, stage of malignancy, TSH level

## Abstract

**Introduction:**

Thyroid cancer is the most common endocrine malignancy, and it has the fastest increase rate in incidence in both sexes, with a yearly increase of 3% over the last decade. Thyroid‐stimulating hormone (TSH) is the main driver for the thyroid gland to produce thyroid hormone. The main purpose of this study was to assess the relationship between serum TSH level and the stage of malignancy in patients with differentiated thyroid cancer.

**Methods:**

This cross‐sectional study was performed on 77 patients with thyroid cancer. The demographic characteristics, TSH level and stage of malignancy were recorded for all patients in the data collection form. The data analysis was conducted by descriptive statistics using SPSS 20.0 software.

**Results:**

The results show a significant relationship (*p*‐value = .025) between the malignancy stage and serum TSH level. The mean TSH level in patients of stage 3 (5.70 ± 2.03) was significantly higher than patients in stage 2 (2.58 ± 0.52) and stage 1 (2.33 ± 0.28). No significant relationship was observed between the age of patients and serum TSH level. Although the mean serum TSH level in men (3.61 ± 0.98) was higher than in women (2.52 ± 0.25), the difference was not statistically significant.

**Conclusions:**

According to the results of this study, serum TSH level can be considered as a predictor of the stage of differentiated thyroid cancer. Therefore, it can be used to predict the likelihood of cancer and improve the outcome and extent of thyroidectomy in patients with thyroid cancer.

## Abstract 2

Thyroid‐stimulating hormone (TSH) is the main driver for the thyroid gland to produce thyroid hormone. The main purpose of this study is to assess the relationship between serum TSH level and the stage of malignancy in patients with differentiated thyroid cancer.

## 1 INTRODUCTION

Thyroid cancer accounts for 1% of all cancer cases worldwide.^1^ Moreover, among all the endocrine malignancies, the highest prevalence and the fastest increase rate in incidence in both sexes is associated with thyroid cancer.^2^ Over 90% of thyroid cancers are differentiated thyroid cancer (DTC), including both papillary thyroid cancer (PTC) and follicular thyroid cancer (FTC).^3^

Presentation of this type of cancer is mostly by nodular goitre, including multinodular goitres and solitary nodules.^4^ Thyroid nodules can be malignant if the patient has signs like rapid growth of nodules, having fixed nodules, hoarseness, dysphagia or lymphadenopathy, history of neck irradiation, male gender and age of younger than 20 years or more than 70 years.^5^, ^6^, ^7^, ^8^

In order to inform DTC management, different staging systems have been developed within the past decades.^9^ American Joint Committee on Cancer (AJCC) proposed the tumour‐node‐metastasis (TNM) staging system for predicting the prognosis of DTC in clinical practice.^10^ In this study, we classified the thyroid cancer cases based on the most updated version of the TNM system (8^th^ edition), which is one of the most popular staging systems across the world. Although understanding the risk factors of thyroid cancer is still controversial,^11^ researchers found a significant relationship between thyroid cancer and exposure to ionizing radiation, family history of thyroid cancer, history of benign thyroid disease, age and gender.^12^, ^13^, ^14^, ^15^

Thyroid‐stimulating hormone (TSH), also known as thyrotropin is the main driver for the thyroid gland to produce thyroid hormone.^16^ In recent years, many studies tried to assess the relationship between the serum TSH level and the risk of thyroid cancer, many of them concluded that higher serum TSH level leads to a higher risk of thyroid cancer.^17^, ^18^, ^19^, ^20^, ^21^, ^22^ Nevertheless, several studies conflicted with this conclusion and reported that no significant relationship was found between thyroid cancer and TSH serum level.^23^ All in all, the relationship between serum TSH levels and thyroid cancer is still poorly understood and not clear,^22^, ^24^, ^25^ Therefore, to gain more insights, we looked at the association between the serum TSH level and stage of malignancy in patients with thyroid cancer.

## 2 MATERIALS AND METHODS

In this prospective study, Clinicopathologic characteristics of 100 patients with confirmed thyroid cancer who were referred to the endocrine clinic in Kerman, Iran in early 2019 were gathered. After reviewing the collected data, we excluded the patients who took any thyroid medication within 3 months prior to the collection of data and those who had incomplete preoperational information. Finally, the total number of 77 patients (12 men and 65 women) were included in this study.

In the process of data collection, various variables were recorded including age, sex, family history of thyroid cancer, history of neck radiotherapy, history of former benign thyroid disease, preoperative serum TSH, Triiodothyronine (T3), fine needle aspiration (FNA) results, histology, size of nodules and surgical pathology report. The study was confirmed by the Ethics Committee of Kerman University of Medical Sciences (permission number: 9600878). The patients were assured that their information will be remained confidential and only being used for research purposes.

### 2.1 Statistical analysis

Statistical relevance of the recorded risk factors for thyroid cancer was calculated by Student's *t*‐test and Pearson chi‐square test for continuous variables and categorical variables respectively. Moreover, we calculated means, standard deviations, frequency and relative frequency for the descriptive statistics. The statistical analysis was conducted using IBM SPSS Statistics 20.0.^26^ In addition, we considered a *p*‐value of less than 0.05 statistically significant.

## 3 RESULTS

In this study, a total number of 77 patients (65 out of which were female) with DTC were staged based on tumour‐node‐metastasis. The age distribution of the subject group shows that 2 (2.5%) patients were under 20 years old, 37 (48.1%) patients were 20–40 years old, 34 (44.2%) patients were 41–60 years old, and 4 (5.2%) patients were over 60 years old.

According to the ultrasound information, 54 (70.1%) patients were with solitary nodules, and 23 (29.8%) patients with multinodular goitre (MNG). Moreover, 5 (6.5%) patients were hyperthyroidism and 11 (14.3%) patients were hypothyroidism. Based on the FNA results, 6 (7.8%) patients were FTC and 71 (92.2%) were PTC. However, the pathology reports show that 5 (6.5%) patients were FTC and 72 (93.5%) were PTC. Based on the TNM stage classification, 73% of the patients were classified as stage I, 20.8% as stage II, 5.2% as grade III and no patient were in stage IV.

As it is illustrated in Table 1, the mean TSH level of patients in stage III (5.70 ± 2.03) is higher than patients in stage I (2.33 ± 0.28) and II (2.58 ± 0.52); in other words, the higher malignancy stages are associated with the higher serum TSH level. Furthermore, the statistical analysis results indicate that there is a significant relationship (*p*‐value = .025) between the malignancy stage and serum TSH level. Nonetheless, no significant relationship (*p*‐value = .781) was identified between patients’ gender and the malignancy stage.

**TABLE 1:** The relationship between malignancy staging (TNM) and TSH level

	Stage I	Stage II	Stage III	*p*‐value
*n* (%)	57 (73%)	16 (20.8%)	4 (5.2%)	.781
Gender (female: *n* (%))	49 (86%)	13 (81.2%)	3 (75%)	
TSH (IU/mL) Mean ± SD	2.33 ± 0.28	2.58 ± 0.52	5.70 ± 2.03	.**025** *

* *p*‐value of less than 0.05 was considered as statistically significant.

The minimum and maximum mean serum TSH levels were associated with the age groups older than 60 years and between 20 and 40 respectively (Table 2). However, the relationship between the age of patients and serum TSH level was not statistically significant (*p* = .920).

**TABLE 2:** The effect of age on malignancy staging and serum TSH level

	Age <20 (years)	Age 20–40 (years)	Age 40–60 (years)	Age >60 (years)	*p*‐value
Stage I (*n* (%))	1 (1.8%)	30 (52.6%)	24 (42.1%)	2 (3.5%)	.364
Stage II (*n* (%))	1 (6.2%)	5 (31.2%)	9 (56.2%)	1 (6.2%)	
Stage III (*n* (%))	0 (0.0%)	2 (50.0%)	1 (25.0%)	1 (25.0%)	
TSH (IU/mL) Mean ± SD	2.25 ± 1.65	2.76 ± 0.38	2.72 ± 0.41	1.96 ± 0.85	.920

Furthermore, the effect of age on malignancy staging is shown in Table 2 where most of the patients within all malignancy stages were 20–60 years old. The statistical analysis indicated that the relationship between age and malignancy staging was not significant (*p* = .364).

Although the mean serum TSH level in men (3.61 ± 0.98) was higher than the mean in women (2.52 ± 0.25), the difference was not statistically significant (Table 3).

**TABLE 3:** The relationship between gender of patients and serum TSH level

Gender	*n* (%)	TSH (IU/mL) Mean ± SD	*p*‐value
Male	12 (15.6%)	3.61 ± 0.98	.135
Female	65 (84.4%)	2.52 ± 0.25	

## 4 DISCUSSION

The relationship between serum TSH level and the malignancy of Thyroid cancer has been confirmed in many studies,^11^, ^27^, ^28^ Haymart et al.^29^ have also shown that when TSH level was less than 0.06 mIU/L the risk of Thyroid cancer was 16%, while it was 52% for the TSH levels of 5.00 mIU/L or greater. Moreover, both frequency and stage of thyroid cancer are perceived to be in association with the serum TSH level.^30^

In the present study, we found a significant relationship between serum TSH level and the stage of malignancy; where the higher stages of malignancy were associated with the higher average serum TSH level. In a study by Haymart et al.^29^ the patients with stage III and IV of malignancy had a mean TSH level of 4.9 ± 1.5 mIU/L, while the patients with stage I and II of the disease had TSH of 2.1 ± 0.2 mIU/L which is much lower.

On the other hand, in a study by Batool et al.^31^ there was no statistically significant association of higher TSH levels with the advanced malignancy stages. In another study by Tam et al.,^1^despite the TSH level was higher in patients with advanced disease stages, the relationship was not considered significant. However, maybe the low number of patients in advanced stages (only 1.3% in stage IV) can explain the conclusion for the latter study. Moreover, He et al.^24^couldn't find a relationship between TSH level and malignancy stage, extrathyroidal invasion, diffusion and prevalence of lymph node metastasis (LNM).

In our study, the mean age of participants was 40.7±11.94 years which does not have a considerable difference with other similar studies,^32^, ^33^ In another study, the malignancy prevalence was found to be significantly higher in patients younger than 40 years old in comparison to the 40–49 year group.^24^ This observation is not consistent with many other cancers that are generally more common in people older than 40 years old. In our study, although the mean age of patients with stage III was higher than the mean age of patients in stage I and II, this difference was not statistically significant (*p* = .364).

In addition, this study showed that women were more likely to be diagnosed with thyroid cancer than men, and this finding is compatible with similar studies,^31^, ^34^ for example in the study by Oberman et al.^34^ 81% of the detected patients were female. However, no significant relationship between gender and stage of malignancy was identified in this study. One of the limitations in our study is the low number of patients in advanced stages (III and IV) that can limit the ability to find a significant relationship between the malignancy stage and age/gender.

In order to assess the patients with thyroid nodules, one of the initial required lab tests is recommended to be the serum TSH level.^35^ Moreover, the TSH level is not only an important factor of thyroid disease but also a beneficial tool for clinical thyroid cancer management.^17^, ^35^, ^36^

Although previous studies have shown that serum TSH level is an independent predictor of thyroid malignancy,^19^ no optimal TSH cut‐off value has been recognized for anticipating the thyroid cancer risk.^17^ Therefore, it is suggested that future studies perform more research on determining an optima TSH cut‐off value to facilitate the use of TSH level as a thyroid cancer predictor. Another gap that can be addressed by future studies is to elaborate more on the effect of age on thyroid malignancy, as the current studies indicate controversial results.

## 5 CONCLUSION

In this study, the association between serum TSH level and differentiated thyroid carcinoma stage was confirmed. Based on the results, no significant relationship between gender and stage of malignancy was identified. Moreover, the difference between the mean age of patients and the malignancy stage was not statistically significant.

## CONFLICT OF INTEREST

The authors declare that they have no conflict of interests.

## AUTHOR CONTRIBUTIONS

**Laya Soleimanisardoo** Data curation‐Lead, Formal analysis‐Lead, Validation‐Equal, Writing‐original draft‐Lead. **Mohsen Rouhani** Formal analysis‐Equal, Software‐Equal, Validation‐Equal, Writing‐review & editing‐Equal. **Fateme Soleymani Sardoo** Formal analysis‐Equal, Software‐Equal, Validation‐Equal, Writing‐review & editing‐Equal. **Mohammad Hossein Gozashti** Conceptualization‐Lead, Methodology‐Lead, Supervision‐Lead, Writing‐review & editing‐Lead.

## ETHICAL APPROVAL

The study was confirmed by the Ethics Committee of Kerman University of Medical Sciences (permission number: 9600878).

## Data Availability

The datasets collected and analysed during the current study are not publicly available due to individual privacy concerns but are available from the corresponding author on reasonable request.
